# Psychometric properties of the Chinese version of the Cognitive Reserve Assessment Scale in Health in patients with cancer

**DOI:** 10.1186/s12888-022-04506-w

**Published:** 2023-01-03

**Authors:** Hong Liu, Yanyan Li, Yang Li, Jianwen Wang, Na Su, Naixue Cui, Kun Xu, Yaoyao Sun, Fenglin Cao

**Affiliations:** 1 Department of Nursing Psychology, School of Nursing and Rehabilitation, Shandong University, No.44 Wenhuaxi Road, Jinan city, 250012 Shandong Province China; 2 School of Nursing, Peking University, Beijing, China; 3 School of Nursing, The University of Texas at Austin, Austin, USA; 4 Imaging Department, Shandong Cancer Hospital and Institute, Jinan, China; 5 Department of Gastrointestinal Surgery, Shandong Cancer Hospital and Institute, Jinan, China

**Keywords:** Cognitive reserve, Cancer, Cognitive function, Cognitive Decline, Validation Study, Psychometrics, China

## Abstract

**Background:**

Cognitive reserve is a modifiable factor that could prevent cognitive decline in patients with cancer. The Cognitive Reserve Assessment Scale in Health (CRASH) is an instrument used to assess cognitive reserve. This study aims to develop and examine the psychometric properties of the Chinese version of the CRASH for patients with cancer.

**Methods:**

A cross-sectional survey was conducted with 167 cancer patients from four wards of two hospitals in China. Thirty-one patients were re-assessed to examine the test-retest reliability. Four translators and three reviewers developed the Chinese version of the scale. We assessed its structural validity, concurrent validity, internal consistency, test-retest reliability, measurement error, and floor/ceiling effects.

**Results:**

Confirmatory factor analysis showed a good model fit with the four-factor structure of the original CRASH. The CRASH scores were statistically significantly associated with neuropsychological test scores, indicating sufficient concurrent validity. The internal consistency was acceptable, except for leisure activities, with standardized Cronbach’s alphas (0.64–0.94) and standardized Omega (0.66–0.95). There was excellent test-retest reliability, with a high intraclass correlation coefficient (0.914–0.993) of total scores and scores for each domain. The measurement error was acceptable, and no floor or ceiling effects were observed*.*

**Conclusions:**

The Chinese version of the CRASH is a valid and reliable instrument to assess cognitive reserve in patients with cancer. Moreover, cognitive reserve measured by the CRASH was associated with low cognitive performance in cancer patients.

## Background

Cancer-related cognitive decline refers to subjective or objective cognitive deterioration in cancer patients after they are diagnosed or receiving treatment; it occurs in approximately 10–50% of cancer patients [1]. Many patients are concerned about the adverse effects of cognitive decline, which may affect their lives and work [2]. The factors that lead to cancer-related cognitive decline are not noticeably clear, and some factors, such as fatigue or genes, are not easily modifiable. It is particularly important to explore factors that can be easily recognized and addressed.

Cognitive reserve, which is deemed as the adaptability of cognitive processes, can facilitate the stability of cognitive performance during pathological or physiological changes in the brain [3]. It can protect against cognitive decline in older adults. By evaluating cognitive reserve, we may be able to identify patients receiving cancer treatment who are at risk for cognitive decline. Concurrently, improving cognitive reserve may be a way to address their cognitive decline. However, few studies have investigated the role of cognitive reserve, and those that have suggest that low cognitive reserve is associated with decreased cognitive function in cancer patients [4, 5]. One of the reasons for the paucity of research on cognitive reserve in cancer patients is the lack of appropriate assessment tools. Previous studies that measured cognitive reserve in cancer survivors used the Wide Range Achievement Test Reading Subscale; however, it is tedious to administer in clinical settings and is not designed to measure cognitive reserve.

Cognitive reserve is viewed as an active process that can be improved through lifetime experiences. These reserve-related factors across the lifespan mainly include educational attainment, occupation, and leisure activities [6]. Some instruments have been developed to measure cognitive reserve by referencing reserve-related factors; however, no Chinese version is available. In addition, some instruments are not suitable for clinical practice or interventions because they are time-consuming, or do not include modifiable factors of cognitive reserve that can be addressed. Furthermore, almost all these tools are designed for older adults, with a lack of measures for younger adults. Yet, cognitive complaints also occur in young cancer patients. The Cognitive Reserve Assessment Scale in Health (CRASH) was developed by Amoretti et al. to assess cognitive reserve in Spanish adults and seniors [7]. It was originally and specifically developed for patients with severe mental illness. It is based on the active model of cognitive reserve [3], including education, occupation, and leisure activities across the phases of life. The CRASH can be used to assess the effectiveness of an intervention in improving cognitive reserve. It takes approximately 10 minutes to complete and can be easily administered by health workers in different professional fields (e.g., nurses, doctors, and psychologists).

The present study aimed to develop the Chinese version of the CRASH by translating it into Chinese and adapting it cross-culturally. In addition, we tested the tool’s psychometric properties including validity (structural validity and concurrent validity), reliability (internal consistency, test-retest reliability, and measurement error), and floor/ceiling effects in patients with cancer, according to the COnsensus-based Standards for the Selection of Health Measurement Instruments checklist (COSMIN) [8]. In the concurrent validity testing, we tried to investigate the associations between objective cognitive function assessed by neuropsychological tests and subjective cognitive function assessed by cognitive reports. We hypothesized that the strength of the association between the CRASH score and objective cognitive function would be greater than that between the CRASH score and subjective function because the subjective cognitive function is more readily influenced by what and how many things cancer patients do in their daily lives.

## Methods

### Sample

A cross-sectional survey was conducted in four departments of two tertiary oncology hospitals in East China from August 2019 to February 2020. The four departments included one radiology department, one medicine department, and two surgery departments. The inclusion criterion was adult or geriatric patients with cancer. Patients who were unable to communicate fluently were excluded. One hundred and sixty-seven hospitalized patients provided informed consent and were recruited for the study. There were 15 patients who declined to participate in our study mainly because they were particularly physically uncomfortable or had a high level of psychological stress. This study was approved by the Ethics Committee of Shandong University School of Nursing and Rehabilitation.

### Development of the Chinese version of the CRASH

The CRASH manual contains semi-structured interviews and a scoring guide. It consists of 33 items and provides a global score and scores for each domain (education, occupation, and leisure activities). Leisure activities include frequency and variety of activities (physical, intellectual, artistic, and cultural), encompassing distinct phases of the participant’s life (childhood/adolescence, adulthood, and the last year). In this domain, sociability across the lifespan was also evaluated. The total score of the CRASH was calculated using the formula: CRASH = (Education × 6 + Occupation × 11.25 + Leisure)/3. The global score ranges from 0–90, with higher scores indicating a better cognitive reserve. The full set of the CRASH items exhibited high internal consistency (α = 0.903) and good construct validity in the Spanish population.

We attained Dr. Amoretti’s permission to translate the CRASH into Chinese and obtained the manual. We translated the scale and interviewing guide of the manual into Chinese, following the translation and cultural adaptation process guidelines [9]. Two Chinese researchers who had a doctoral degree and master’s in health psychology conducted the forward translation. Subsequently, these two researchers discussed their translations with a professor possessing a doctoral degree in psychology and expertise in instrument development. An agreement was reached on the following points: (1) schooling question: the academic qualifications and corresponding scoring were revised to adapt to the Chinese context (0 = primary school or below, 1 = junior high school, 2 = high school or secondary school, 3 = higher vocational schools, 4 = undergraduate, 5 = master or doctor); (2) school performance during childhood and adolescence: the 10-point system often used for exams in Western countries was replaced by the 100-point system commonly used in China; and (3) discrepancies in understanding between the two translators were solved through discussion with the professor. Thereafter, two other translators translated the Chinese version back into English. Both translators had doctoral degrees in health psychology; one studied in China and the USA and the other had a master’s degree in English. Subsequently, the four translators, a professor, and a reviewer with a doctoral degree in health psychology compared all translations with the original and reconciled the remaining discrepancies. After reaching a consensus, a single Chinese version was developed. The Chinese version was reviewed by a Chinese reviewer with a doctoral degree in health psychology who had worked at an American university for five years. Minor modifications in semantics were made to ensure it was more humane and accessible to participants and interviewers. Three postgraduate nursing students and five oncology nurses used the final Chinese version to interview patients with cancer. They provided feedback to the researchers on the interpretability and understandability of the Chinese version of the scale.

### Assessments

#### Objective cognitive function

Several neuropsychological tests were selected according to recommendations for cancer patients, developed by the International Cognition and Cancer Task Force [10]. The auditory verbal learning test (AVLT) [11] includes AVLT short-term memory (AVLT-I) and AVLT delayed recall (AVLT-II), which were used to assess participants’ vocabulary, learning ability, and delayed memory, respectively. The shape trail test (STT) [12], a modified version of the Trail Making Test for Chinese people, is composed of STT-A and STT-B. Processing speed was tested with the STT-A, and executive function was assessed using the STT-B. Speeded lexical fluency was tested using the verbal fluency test (VFT) [13]. The Wechsler Adult Intelligence Scale-digit span test (WAIS DST) [14] contains DST-forward and DST-backward to evaluate attention and working memory, respectively. Higher scores on all tests, except the STT, indicate better cognitive function.

#### Subjective cognitive function

Subjective cognitive function was evaluated using Functional Assessment of Cancer Therapy-Cognitive Function (FACT-Cog) version 4.0. The FACT-Cog contains four subscales: perceived cognitive impairments (PCI), perceived cognitive abilities (PCA), comments from others, and the impact of cognitive changes on the quality of life [15]. The Chinese version showed good internal consistency and test-retest reliability [16]. Patients’ cognitive impairment and ability were the focus of this study, therefore we only included PCI and PCA [17]. Higher scores indicated worse subjective cognitive function.

#### Other assessments

The Functional Assessment of Chronic Illness Therapy-Fatigue (FACIT-F) was used to assess fatigue [18]. Depressive symptoms were evaluated using the 9-item Patient Health Questionnaire (PHQ-9). The Generalized Anxiety Disorder Screener (GAD-7) was used to assess patients’ anxiety symptoms [19].

### Data collection

A trained researcher collected the data. The researcher used a face-to-face approach to pose assessment questions to the patients. To determine the test-retest reliability of the CRASH, the researcher used convenience sampling to select 31 patients [20] when they returned to the hospital for treatment, two or three weeks after their initial assessment [21], for re-assessment.

### Statistical analysis

SPSS v25.0 (IBM Corp., Armonk, NY, USA) and Mplus Version 8.3 were used to conduct statistical analyses. A 5% significance level was adopted. Descriptive statistics (means ± standard deviations, range) were generated for continuous variables, and frequencies and percentages were calculated for categorical variables. The reliability and validity of the CRASH were evaluated according to the COSMIN [22], a guideline for studies on measurement properties of health-related patient-reported outcomes.

#### Structural validity

The Kaiser–Meyer–Olkin (KMO) and Bartlett’s test of sphericity were performed to ensure our assumptions associated with factor analysis were met. Confirmatory factor analysis (CFA) was conducted to cross-validate the original four-component structure reported by the CRASH development study. This structure included education, occupation, sociability, and leisure activities. The CFA model fit was identified as good if the root mean squared error of approximation (RMSEA) was lower than 0.08, standardized root mean square residual (SRMR) was lower than 0.06, and comparative fit index (CFI) and Tucker Lewis Index (TLI) were higher than 0.95 [23].

#### Concurrent validity

Spearman’s correlations between the scores of the CRASH, neuropsychological tests (AVLT-I, AVLT-II, STT-A, STT-B, DST, and VFT), and cognitive reports (PCI and PCA of FACT-Cog) were conducted to test the concurrent validity of the Chinese version of the CRASH (effect size: r = 0.10–0.29 as small, r = 0.30–0.49 as medium, and r ≥ 0.50 as large) [24]. Multiple linear regression using the enter method was conducted to examine whether cognitive reserve could predict the cognitive function of patients with cancer. In the regression model, scores from the neuropsychological tests (including AVLT-I, AVLT-II, STT-A, STT-B, DST, and VFT) and cognitive reports (PCI and PCA of FACT-Cog) were included as dependent variables. Scores of the CRASH and each domain were included as independent variables. We controlled for age, gender, and factors that can influence cognitive function as independent variables including fatigue and depressive and anxiety symptoms which were assessed by FACIT-F, PHQ-9, and GAD-7, respectively.

To investigate the association between current social and leisure activities and cognitive function, which is a modifiable component of cognitive reserve, two additional multiple linear regressions using the enter method were conducted. We used the scores of current sociability and leisure activities as independent variables and cognitive function as the dependent variable. We controlled for age, gender, fatigue, depressive symptoms, and anxiety symptoms.

#### Internal consistency

Each dimension of the CRASH had a different weighting. We therefore calculated standardized Cronbach’s alpha and McDonald’s omega for each domain and the entire CRASH.

#### Test-retest reliability

A two-way mixed-effects model was conducted to calculate the intraclass correlation coefficient (ICC), where an ICC of 0.7 or larger indicated preferable test-retest reliability [25].

#### Measurement error

The standard error of measurement (SEM) was calculated as $\sqrt{\upsigma \textrm{random}}$, and the smallest detectable change (SDC) was determined according to the formula SDC = 1.96×$\sqrt{2}$×SEM [26]. An SDC < 10% total score of the CRASH was considered acceptable [27].

#### Floor or ceiling effects

Floor or ceiling effects were deemed positive if > 15% of the participants had the lowest or highest CRASH scores, respectively [28].

## Results

### Participants characteristics

A total of 182 patients were eligible for our study, and 167 patients who were planning to have surgery or adjuvant treatment provided informed consent. The mean age of the participants was 58.2 years, with a standard deviation of 9.7 years, and their ages ranged from 30–79 years. There were 128 participants aged < 65 years, accounting for 76.6% of the total sample. There were 128 participants (76.6%) aged < 65 years, and 139 (83.2%) participants were born before 1970. Most of the participants were male (65.9%), married (99.4%), and currently living in the city (73.7%). There were 31 participants whose conditions were stable and who completed the reassessment upon returning for treatment. The average age of the retested participants was 57.8±9.5 years, with a range from 34 to 77 years. They were all married. The demographics and clinical characteristics of the baseline sample and retested sample are shown in Table 1.

**Table 1:** Characteristics of Baseline and Retested Samples

		Baseline sample (*n* = 167)	Retested sample (*n* = 31)
		M±SD/n (%)	M±SD/n (%)
Age		58.2±9.6	57.8±9.5
Gender	male	110 (65.9%)	12 (38.7%)
	female	57 (34.1%)	35 (35.4%)
Residence	city	123 (73.7%)	30 (96.8%)
	rural	44 (26.3%)	1 (3.2%)
Cancer	colorectal	28 (16.8%)	2 (6.5%)
	rectal	44 (26.3%)	2 (6.5%)
	gastric	37 (22.2%)	8 (25.8%)
	thyroid	10 (6.0%)	6 (19.4%)
	other	48 (28.7%)	13 (41.9%)
Cancer periods	I	16 (9.6%)	2 (6.5%)
	II	50 (29.9%)	6 (19.4%)
	III	65 (38.9%)	10 (32.3%)
	IV	36 (21.6%)	13 (41.9%)
Past operation	yes	67 (40.1%)	13 (41.9%)
	no	100 (59.9%)	18 (58.1%)
Past chemotherapy	yes	60 (35.9%)	19 (61.3%)
	no	107 (64.1%)	12 (38.7%)
Past targeted therapy	yes	13 (7.8%)	5 (16.1%)
	no	154 (92.2%)	26 (83.9%)
Past radiotherapy	yes	24 (14.4%)	6 (19.4%)
	no	143 (85.6%)	25 (80.6%)

The CRASH total scores ranged from 3.75–62.73, with a mean ±standard deviation of 25.92±11.14. Among the 167 patients, 68 completed all the neuropsychological tests and FACT-Cog. Only 76–80 patients completed the STT-B, DST, and VFT because we wanted to reduce the burden on patients later. Ten patients declined to undergo tests due to mood or illiteracy. Demographics and clinical characteristics of participants who completed all the measures and who only completed part of the measures are shown in Table 2.

**Table 2:** Characteristics of Baseline Information in Participants who Completed All the Measures versus Some of the Measures

		All measures completed (*n* = 68)	Some measures completed (*n* = 89)	t/χ^2^	*P*
		M±SD/n (%)	M±SD/n (%)		
Age		58.7±10.5	57.8±9.0	0.587	0.558
Gender	male	46 (67.6%)	57 (64.0%)	0.222	0.638
	female	22 (32.4%)	32 (36.0%)		
Residence	city	55 (80.9%)	63 (70.8%)	2.104	0.147
	rural	13 (19.1%)	26 (29.2%)		
Cancer	colorectal	9 (13.2%)	19 (21.3%)	1.963	0.743
	rectal	18 (26.5%)	22 (24.7%)		
	gastric	15 (22.1%)	16 (18.0%)		
	thyroid	22 (32.4%)	26 (29.2%)		
	other	4 (5.9%)	6 (6.7%)		
Cancer periods	I	5 (7.4%)	11 (12.4%)	7.338	0.062
	II	23 (33.8%)	25 (28.1%)		
	III	31 (45.6%)	28 (31.5%)		
	IV	9 (13.2%)	25 (28.1%)		
Past operation	yes	23 (33.8%)	39 (48.3%)	1.612	0.204
	no	45 (66.2%)	50 (56.2%)		
Past chemotherapy	yes	22 (32.4%)	35 (39.3%)	0.810	0.368
	no	46 (67.6%)	54 (60.7%)		
Past targeted therapy	yes	4 (5.9%)	9 (10.1%)	0.908	0.341
	no	64 (94.1%)	80 (89.9%)		
Past radiotherapy	yes	8 (11.8%)	16 (18.0%)	1.149	0.284
	no	60 (88.2%)	73 (82.0%)		

The results suggest that the baseline characteristics were not significantly different. The results of the neuropsychological performance and cognitive reports are shown in Table 3.

**Table 3:** Neuropsychological Performance and Cognitive Reports of Cancer Patients

	*N*	Mean ± SD	range
AVLT-I	72	13.46±4.56	5–23
AVLT-II	72	3.54±2.10	0–8
STT-A	68	72.79±49.28	23–404
STT-B	154	224.49±120.18	53–928
VFT	152	16.93±5.51	3–33
DST-forward	156	7.45±1.31	5–11
DST-backward	156	3.72±1.24	0–9
PCI	76	13.25±11.77	0–46
PCA	76	11.71±5.22	4–25

*Abbreviations*: *SD* Standard deviation, *AVLT-I* Auditory Verbal Learning Test -I- short-term memory, *AVLT-II* Auditory Verbal Learning Test -II- delayed recall, *STT-A* Shape Trail Test -A- processing speed, *STT-B* Shape Trail Test-B- executive function, *VFT* Verbal Fluency Test, *DST-forward* Digit Span Test to evaluate attention, *DST-backward* Digit Span Test to evaluate working memory, *PCI* Perceived cognitive impairments, *PCA* Perceived cognitive abilities

.

### Structural validity

**Fig. 1:**
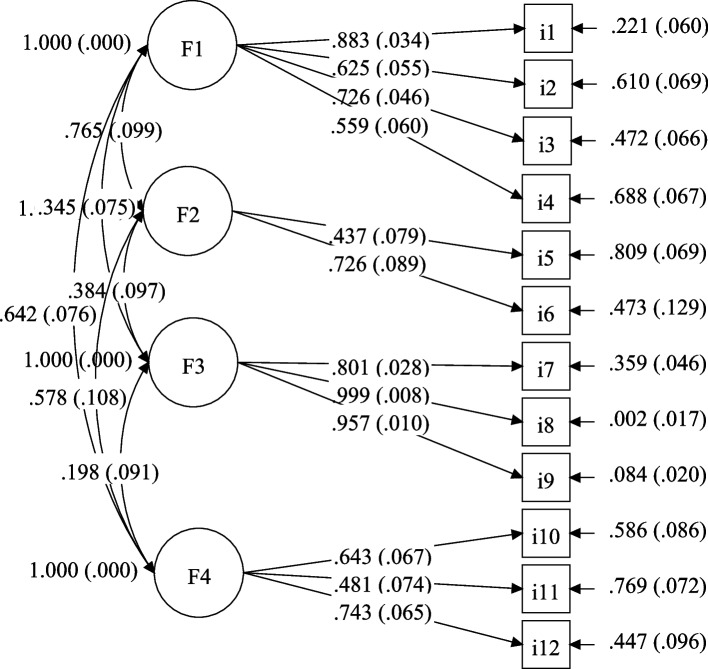
The model's standardized parameter estimates obtained through confirmatory factor analysis. *Notes.* Domains: F1, education; F2, occupation; F3, sociability; F4, leisure activities

The results of Bartlett’s test of sphericity showed that our data was adequate for factor analysis (χ^2^ = 1045.171, df = 66, *p*<.001; Kaiser-Meyer-Olkin = 0.774). The CFA results showed that the model fit was good, RMSEA = 0.064 (< 0.08), SRMR = 0.048 (< 0.06), CFI = 0.968 (> 0.95), and TLI = 0.956 (> 0.95). The standardized estimates of factor loadings were all higher than 0.4 with *p* < .001, indicating the structure was acceptable. Fig. 1 presents the standardized parameter estimates of the model conducted by CFA, including the standard factor loadings and corresponding standard errors, residual variances, corresponding standard errors, and correlations between four factors.

### Concurrent validity

There were small to medium correlations between the CRASH total scores and neuropsychological performances, except for STT-A. Education had a medium relationship with the scores from all neuropsychological tests. Occupation and leisure activities had small to medium correlations with STT-B, DST-forward, DST-backward, and VFT. Sociability was the only factor that had a positive relationship with AVLT-I. Correlations between cognitive reports and CRASH total scores and scores for each domain were not statistically significant (Table 4).

**Table 4:** Spearman Correlation Analyses of the CRASH and Cognitive Functions

	Education	Occupation	Sociability	Leisure Activities	Total
AVLT-I	0.367^**^	0.197	0.343^**^	0.121	0.301^*^
AVLT-II	0.343^**^	0.168	0.211	0.077	0.245^*^
STT-A	-0.342^**^	-0.102	-0.214	-0.043	-0.200
STT-B	-0.467^***^	-0.248^**^	-0.089	-0.218^**^	-0.392^***^
VFT	0.423^***^	0.273^**^	0.052	0.310^***^	0.393^***^
DST-forward	0.380^***^	0.333^***^	0.143	0.244^**^	0.403^***^
DST-backward	0.395^***^	0.335^***^	0.122	0.231^**^	0.396^***^
PCI	0.003	0.035	-0.036	0.044	0.054
PCA	-0.066	0.035	-0.106	-0.039	-0.005

*Abbreviations*: *AVLT-I* Auditory Verbal Learning Test -I- short-term memory, *AVLT-II* Auditory Verbal Learning Test -II- delayed recall, *STT-A* Shape Trail Test -A- processing speed, *STT-B* Shape Trail Test-B- executive function, *VFT* Verbal Fluency Test, *DST-forward* Digit Span Test to evaluate attention, *DST-backward* Digit Span Test to evaluate working memory, *PCI* Perceived cognitive impairments, *PCA* Perceived cognitive abilities

^*^p < .05, ^**^p < .01, ^***^p < .001, two-tailed

A higher CRASH total score was statistically significantly associated with better performance on AVLT-I, STT-B, DST-forward, DST-backward, and VFT in multiple linear regression models, controlling for age, gender, fatigue, and depressive and anxiety symptoms. Education was statistically significantly associated with neuropsychological performance, and occupation was associated only with DST-backward performance. Better sociability was associated with better performance on all neuropsychological tests, except for DST-backward. Leisure activities were statistically significantly associated with STT-B, DST-backward, and VFT performance. No statistically significant associations were observed between cognitive reports, total CRASH scores, and scores for each domain (Table 5).

**Table 5:** Multiple Linear Regression Analyses of Associations between CRASH and Cognitive Function

Cognitive function	*N*	Education		Occupation		Sociability		Leisure Activities		Total	
		*β*	*t*	*β*	*t*	*β*	*t*	*β*	*t*	*β*	*t*
AVLT-I	69	0.30	2.82^**^	0.15	1.35	0.42	4.12^**^	0.13	1.11	0.28	2.64^*^
AVLT-II	69	0.23	2.03^*^	0.10	0.87	0.26	2.30^*^	0.02	0.15	0.18	1.65
STT-A	65	-0.25	-2.27^*^	-0.06	-0.48	-0.40	-3.79^***^	-0.16	-1.42	-0.21	-1.93
STT-B	65	-0.30	-3.09^**^	-0.14	-1.32	-0.40	-4.25^***^	-0.28	-2.83^**^	-0.30	-3.12^**^
VFT	65	0.41	4.24^***^	0.20	1.89	0.25	2.28^*^	0.37	3.68^**^	0.39	3.98^***^
DST-forward	65	0.31	2.87^**^	0.22	1.96	0.27	2.39^*^	0.12	0.99	0.30	2.77^**^
DST-backward	67	0.34	3.01^**^	0.25	2.12^*^	0.21	1.69	0.24	2.01^*^	0.34	3.07^**^
PCI	76	-0.07	-0.68	-0.07	-0.63	-0.09	-0.86	-0.09	-0.90	-0.09	-0.90
PCA	76	-0.12	-1.17	-0.05	-0.54	-0.08	-0.77	-0.16	-1.57	-0.12	-1.24

*Abbreviations*: *AVLT-I* Auditory Verbal Learning Test -I- short-term memory, *AVLT-II* Auditory Verbal Learning Test -II- delayed recall, *STT-A* Shape Trail Test -A- processing speed, *STT-B* Shape Trail Test-B- executive function, *VFT* Verbal Fluency Test; DST-forward, Digit Span Test to evaluate attention, *DST-backward* Digit Span Test to evaluate working memory, *PCI* Perceived cognitive impairments, *PCA* Perceived cognitive abilities. Controlling for age, gender, fatigue, and depressive and anxiety symptoms

^*^p < .05, ^**^p < .01, ^***^p < .001, two-tailed.

In our further analysis of multiple linear regression, the results remained almost the same as those in the primary analysis. Except for DST-backward, better sociability in the past year was associated with better performance on all neuropsychological tests. Abundant leisure activities in the past year were statistically significantly associated with STT-B and VFT performance. No statistically significant associations were observed between cognitive reports, sociability, and leisure activities in the past year (Table 6).

**Table 6:** Multiple Linear Regression Analyses of Associations between Cognitive Function and Sociability and Leisure Activities in the Past Year

Cognitive function	*N*	Sociability in the past year		Leisure Activities in the past year	
		β	*t*	β	*t*
AVLT-I	69	0.43	4.25^***^	0.19	1.68
AVLT-II	69	0.28	2.48^*^	0.04	0.35
STT-A	65	-0.38	-3.51^**^	-0.20	-1.69
STT-B	65	-0.38	-4.07^***^	-0.25	-2.47^*^
VFT	65	0.27	2.43^*^	0.34	3.24^**^
DST-forward	65	0.29	2.54^*^	0.09	0.78
DST-backward	67	0.22	1.77	0.16	1.32
PCI	76	-0.08	-0.76	-0.03	-0.25
PCA	76	-0.07	-0.67	-0.15	-1.48

*Abbreviations*: *AVLT-I* Auditory Verbal Learning Test -I- short-term memory, *AVLT-II* Auditory Verbal Learning Test -II- delayed recall, *STT-A* Shape Trail Test -A- processing speed, *STT-B* Shape Trail Test-B- executive function, *VFT* Verbal Fluency Test, *DST-forward* Digit Span Test to evaluate attention, *DST-backward* Digit Span Test to evaluate working memory, *PCI* Perceived cognitive impairments, *PCA* Perceived cognitive abilities. Controlling for age, gender, fatigue, and depressive and anxiety symptoms

^*^p < .05, ^**^p < .01, ^***^p < .001, two-tailed

### Internal consistency

The internal consistency of the CRASH was examined using both standardized Cronbach’s alpha and Omega. The standardized Cronbach’s alpha was 0.83 for the entire CRASH, and domain scores were: 0.80 for education, 0.64 for occupation, 0.94 for sociability, and 0.65 for leisure activities. Standardized McDonald's omega was 0.70 for the entire CRASH, and domain scores were as follows: 0.80 for education, 0.77 for occupation, 0.95 for sociability, and 0.66 for leisure activities.

### Test-retest reliability

Thirty-one participants completed the re-assessment. The ICC values of each domain and total CRASH were larger than 0.9. The ICC value for the CRASH total score was 0.985 (95% CI: 0.969–0.993, *p* < .001); education factor was 0.978 (95% CI: 0.954–0.989, *p* < .001); occupation factor was 0.914 (95% CI: 0.829–0.957, *p* < .001); sociability factor was 0.972 (95% CI: 0.942–0.986, *p* < .001), and leisure activities was 0.993 (95% CI: 0.986–0.997, *p* < .001).

### Measurement error

The σ_random_ of the CRASH was 3.142. According to this formula, the SEM was 1.773, and the SDC was 4.914. CRASH total scores ranged from 0–90; the measurement error was acceptable and the SDC was less than 10% of the total scores [27].

### Floor or ceiling effects

No patients reached the minimum (0) or maximum score (90), indicating the absence of floor or ceiling effects.

## Discussion

In the present study, we translated the CRASH from English into Chinese and adapted it for the Chinese context. We examined the tool’s psychometric properties with reference to the COSMIN by administering the scale to patients with cancer. Our results showed sufficient validity (structural and concurrent validity), good reliability (internal consistency, test-retest reliability, and measurement error), and no floor or ceiling effects.

The CFA on the Chinese CRASH items assessed the fit of the measurement model developed initially by Amoretti et al. [7] using the Spanish CRASH, and the model fit indices showed that the fit was good. In addition, the standard factor loadings of all items were above 0.4 with *p* < .001, indicating that the items grouped into the four domains can be considered acceptable.

Our results for concurrent validity suggested that a higher total score of the CRASH was associated with better performance on AVLT-I, STT-B, DST-forward, DST-backward, and VFT when controlling for factors related to cognitive function. This was consistent with the results of the original English CRASH that the score of the English version was associated with the performance of neuropsychological tests on attention, verbal fluency, verbal memory, and processing speed [29]. The correlation analysis showed that the total score of the CRASH had a medium relationship with executive function, verbal fluency, attention, and working memory and a small relationship with short-term memory and delayed recall. Among the CRASH domains, education was the most related to cognitive performance and had a medium relationship with all neuropsychological test scores. Occupation had a small relationship with executive function and verbal fluency and a medium relationship with attention and working memory. Sociability had a medium relationship with short-term memory. Leisure activities had a small relationship with executive function, attention, and working memory and a medium relationship with verbal fluency.

Unlike the original scale, we added a multiple linear regression analysis to demonstrate that cognitive reserve measured by the Chinese CRASH is still associated with cognitive function after controlling for factors that affect cognitive function. The results of the regression models showed that when controlling for age, gender, fatigue, depression, and anxiety, the CRASH total score was associated the most with verbal fluency (β=0.39, *P*<0.001) and was also significantly associated with short-term memory, delayed recall, executive function, attention, and working memory. Among the CRASH domains, education and sociability were mostly associated with cognitive function. Education was more associated with verbal fluency (β = 0.41, *p* < 0.001) than other cognitive functions, as were leisure activities (β = 0.37, *p* < 0.01). Sociability was most significantly associated with short-term memory (β = 0.42, *p* < 0.01) and had relatively large associations with processing speed (β = -0.40, *p* < 0.001) and executive function (β = -0.40, *p* < 0.001). Occupation appeared to be the least associated with cognitive function and only with working memory. Modifiable factors of the CRASH, such as current leisure activities and sociability, were associated with different aspects of neuropsychological performance. Current sociability was mostly associated with short-term memory (β = 0.43, *p* < 0.001), attention (β = -0.38, *p* < 0.01), and executive function (β = -0.38, *p* < 0.001). Yet, current leisure activities tended to be associated with verbal fluency (β = 0.34, *p* < 0.01). Thus, interventions developed to enhance cognitive reserve modifiable factors may prevent or delay cognitive decline in patients with cancer. However, no statistically significant associations were found between the CRASH scores and subjective cognitive function (PCI and PCA). The discrepancy in performance between subjective and objective cognitive function in cancer survivors was reported in several studies [30]. One possible explanation for our results is that subjective cognitive function is influenced to a certain extent by the difficulty and quantity of things that individuals usually do. Many participants informed us that they did not need to be involved in home or work activities as their families encouraged them to rest. They may not subjectively notice changes in their cognitive function due to uncomplicated daily activities. However, neuropsychological tests could reveal impaired cognitive function when patients must perform complicated tasks.

The reliability of the Chinese version of the CRASH was good, as demonstrated by acceptable internal consistency, excellent test-retest reliability, and acceptable measurement error [22]. Regarding the internal consistency, the standardized Cronbach’s alpha and standardized McDonald's omega of the whole CRASH (0.83 and 0.70, respectively), domains of education (0.80 and 0.80, respectively), and sociability (0.94 and 0.95, respectively) were good. The Cronbach’s alpha for the occupation domain (0.64) seemed to be slightly low according to the widely accepted alpha value of 0.70. This may be because the number of occupation items (two) was too small to calculate a high Cronbach’s alpha. The Cronbach’s alpha is easily affected by the number of items, and the fact that Cronbach’s alpha was smaller due to fewer items does not mean that the internal consistency is low. In fact, McDonald's omega is not affected by the number of items, and the omega value of 0.77 for the occupation domain suggested good internal consistency. Both the standardized Cronbach’s alpha and McDonald's omega of the leisure activities domain were somewhat small (0.65 and 0.66, respectively). The reason for this may be the differences in participation between life stages, especially between childhood and adulthood. Although the internal consistency value, which was less than 0.70 but more than 0.65, was acceptable in some literature [31], it needs to be further tested in a larger sample. Therefore, in general, the internal consistency of the Chinese CRASH is acceptable except for the leisure activities domain which is a little low. As for the original English CRASH, the authors only tested the internal consistency of the whole CRASH with the Cronbach’s alpha being 0.903, which was higher than the Chinese one. Furthermore, the ICC values of the total scale and each domain (0.914–0.993) showed excellent test-retest reliability [25]. The measurement error was acceptable, as the SDC (4.914) was smaller than nine (10% of the CRASH total score) [27]. Finally, the CRASH showed no floor or ceiling effects in our sample.

However, there are limitations to our study. First, the internal consistency of the CRASH leisure activities domain is somewhat low. Second, we did not examine the convergent validity or known-groups validity as there were no other Chinese instruments to assess cognitive reserve, and this also made it impossible to compare the relevant results to the original CRASH. In addition, unlike psychiatric disorders, the disease or therapeutic factors that can distinguish between high and low cognitive reserve are unclear, which also limited us from assessing the known-groups validity. Third, we did not examine the inter-rater reliability or sensitivity to change, which must be remedied in the future validation of the scale. Fourth, most of the participants grew up when China's economy was in bad shape (born before 1970), but the scale needs to be validated in a larger sample with a younger population as China's economy has grown exponentially in recent years and individual’s education and leisure patterns have changed dramatically. Fifth, we did not collect demographic or clinical information on the patients who refused to participate in our study and could not compare whether there were differences in demographic or clinical characteristics between them. The fact that the patients refused to participate in our study mainly because of physical or psychological discomfort reflects a potential selection bias that we may have had in recruiting participants.

## Conclusions

The Chinese version of the CRASH is a valid and reliable instrument to assess cognitive reserve in patients with cancer. Moreover, the results suggest that cognitive reserve measured by the CRASH was associated with low cognitive performance in cancer patients.

## Data Availability

The datasets generated and analysed during the current study are not publicly available because we do not consent from all patients to publish this data and this data involves patient privacy but are available from the corresponding author on reasonable request. The Chinese version of the CRASH can be obtained from the corresponding author or the scale developer.
